# Estimating influenza outpatients' and inpatients' incidences from 2009 to 2011 in a tropical urban setting in the Philippines

**DOI:** 10.1111/irv.12223

**Published:** 2014-01-03

**Authors:** Veronica L Tallo, Taro Kamigaki, Alvin G Tan, Rochelle R Pamaran, Portia P Alday, Edelwisa S Mercado, Jenaline B Javier, Hitoshi Oshitani, Remigio M Olveda

**Affiliations:** a Department of Epidemiology and Biostatistics, Research Institute for Tropical Medicine, Department of HealthManila, Philippines; b Department of Virology, Tohoku University Graduate School of MedicineSendai, Japan; c Research Institute for Tropical Medicine, Department of HealthManila, Philippines; d Molecular Biology Laboratory, Research Institute for Tropical Medicine, Department of HealthManila, Philippines

**Keywords:** Disease burden, influenza, influenza-like illness, Philippines, severe acute respiratory infection

## Abstract

**Objectives:**

Although the public health significance of influenza in regions with a temperate climate has been widely recognized, information on influenza burden in tropical countries, including the Philippines, remains limited. We aimed to estimate influenza incidence rates for both outpatients and inpatients then characterized their demographic features.

**Design:**

An enhanced surveillance was performed from January 2009 to December 2011 in an urbanized highland city. The influenza-like illness (ILI) surveillance involved all city health centers and an outpatient department of a tertiary government hospital. The severe acute respiratory infection (sARI) surveillance was also conducted with one government and four private hospitals since April 2009. Nasal and/or oropharyngeal swabs were collected and tested for influenza A, influenza B, and respiratory syncytial virus.

**Results and Conclusions:**

We obtained 5915 specimens from 13 002 ILI cases and 2656 specimens from 10 726 sARI cases throughout the study period. We observed year-round influenza activity with two possible peaks each year. The overall influenza detection rate was 23% in the ILI surveillance and 9% in the sARI surveillance. The mean annual outpatient incidence rate of influenza was 5·4 per 1000 individuals [95% confidence interval (CI), 1·83–12·7], and the mean annual incidence of influenza-associated sARI was 1·0 per 1000 individuals (95% CI, 0·03–5·57). The highest incidence rates were observed among children aged <5 years, particularly those aged 6–23 months. Influenza posed a certain disease burden among inpatients and outpatients, particularly children aged <5 years, in an urbanized tropical city of the Philippines.

## Background

The global impact of influenza is mirrored by the efforts of several countries to conduct and set up influenza surveillance networks and studies to define its impact.^1^–^3^ Although the public health significance of influenza has been recognized globally, studies measuring influenza burden are sparse in developing countries. The influenza disease burden is usually assessed in terms of mortality, morbidity, and economic loss. These estimations require a rigorous study design and research capacity.^4^

The Philippines is located in a tropical climate zone. It is classified as a lower–middle income country by the World Bank.^5^ Respiratory infections, including influenza, are important causes of morbidity and mortality in the country.^6^ Despite the potential public health impact of influenza, there is currently no national policy on influenza prevention and control in the Philippines. The Research Institute for Tropical Medicine (RITM) has been operating as the National Influenza Center (NIC) of the country since 2004. It has set up a surveillance network comprising two sentinel sites in 12 of its 17 administrative regions and has also initiated severe acute respiratory infection (sARI) surveillance. Despite this progress, some gaps in our knowledge still remain, particularly with regard to the burden of influenza in the country. This study aims to describe the epidemiology of influenza and estimate the incidences of influenza outpatients and inpatients between 2009 and 2011 in an urbanized tropical city of the Philippines.

## Materials and methods

### Study site

Baguio City is an urbanized highland city in northern Luzon. Despite the monthly average temperature ranging from 17 to 22°C, the city has the same climate pattern dominant in the rest of Luzon Island, with two pronounced seasons: the dry season from November to May and the wet season during the rest of the year. The 2007 census revealed a population of 301 900, and two-thirds of the population was aged <30 years.

Health services are provided through the 16 health centers of the Baguio Health Department, where primary health care is dispensed by physicians, nurses, and midwives. Each health center covers approximately 5000 populations. A physician assigned to these facilities attends to morbidity consultations and the administration of other medical programs on specific days. There is also one government hospital and five private hospitals with bed capacities of 10–250 beds; these provide inpatient care for the city population.

This study was reviewed and approved by the RITM Institutional Review Board on March 31, 2009.

### Enhanced influenza-like illness surveillance

**Figure 1:**
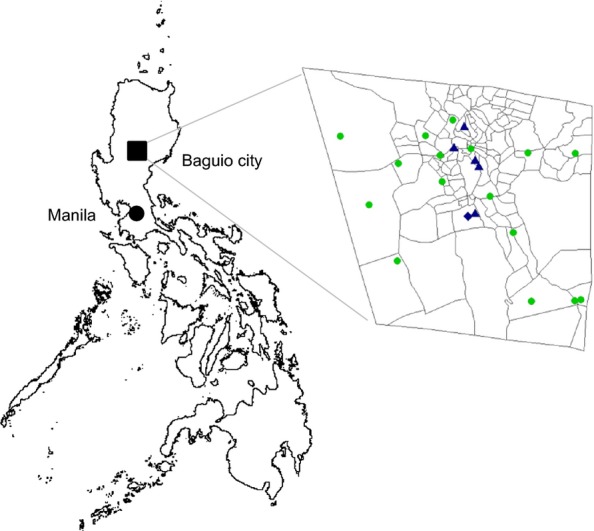
Location map of Baguio city in the Philippines (A). Participating health centers and hospitals in Baguio city (B). The filled circles indicate 16 city health centers, the filled triangles indicate hospitals, and the diamonds indicate hospitals covering both ILI and sARI surveillance. ILI, influenza-like illness; sARI, severe acute respiratory infection.

Enhanced influenza-like illness (ILI) surveillance was conducted in all 16 health centers and the outpatient department of the government hospital (Figure 1). An ILI case was defined as one who developed sudden onset of fever over 38°C with cough or sore throat. Influenza surveillance nurses (ISNs) were allocated on one of two morbidity consultation days when a physician attends and they compiled demographic, clinical, and epidemiological information into the standard case report forms and collected naso- or oropharyngeal swabs. Influenza surveillance nurses also collected the data of ILI cases who visited other than that specific surveillance day in preceding week. Refusal to participate in the surveillance was low at 1·1%, 0·5%, and 1·8% for 2009, 2010, and 2011, respectively.

### Severe acute respiratory infection surveillance

A sARI case was defined as one with ILI accompanied by shortness of breath or difficulty in breathing who required hospital admission. Among children, pneumonia or severe pneumonia was diagnosed on the basis of the Integrated Management of Child's Illness (IMCI) algorithm.^7^ Symptoms included cough and difficulty in breathing and at least one of the following danger signs: chest indrawing, stridor while calm, history of convulsions, inability to drink, and lethargy.

The ISNs approached all patients with a diagnosis of sARI on admission and daily reviewed their hospital admission records until hospital discharge to determine outcomes. Demographic, clinical, and epidemiological information was obtained using a structured questionnaire. Naso- or oropharyngeal swabs were also collected. The surveillance commenced in six hospitals from April 2009, but eventually included five hospitals by 6 months after study initiation because one hospital shut down. Refusal rates for the sARI surveillance were 2%, 5%, and 7% for 2009, 2010, and 2011, respectively.

### Laboratory methods

Naso- or oropharyngeal swabs were obtained from each case using Dacron swabs. The specimens were stored in virus transport media with ice packs to maintain a refrigerated temperature after collection and transported to the NIC. All specimens were tested for influenza A by real-time reverse transcriptase polymerase chain reaction (RT-PCR) using the United States Centers for Disease Control and Prevention and Control (CDC) method^8^ or the Applied Biosystems Pandemic H1N1/09 Assay Set v 2.0 (Life Technologies, Carlsbad, CA, USA). Influenza A-positive samples were further subtyped for influenza A (H1N1) pdm09 (H1N1pdm09) using the CDC method^8^ or the Pandemic H1N1/09 Assay Set v 2.0, starting from May 2009. Seasonal influenza A (H1N1) and influenza A (H3N2) were detected by using the Centre for Health Protection, Department of Health, Hong Kong SAR method.^9^ Influenza B and respiratory syncytial virus (RSV) were detected using a previously published conventional RT-PCR method.^10^

### Data analyses

The virus positivity rate was calculated as the proportion of the number of positive samples to the total number of samples tested. The government's population projection, based on the 2007 census data, was used to estimate the age-specific population. We estimated the influenza incidence rate, expressed as per 1000 individuals by each age group, by dividing the number of patients with ILI who presented at the health facilities and whose samples tested positive for influenza by the census population, followed by multiplying with the inverse of the proportion of the number of samples obtained to the total ILI count. A similar procedure was followed for estimating RSV and influenza-associated sARI incidence rates. We estimated the seasonal influenza incidence rate, including seasonal H1N1, H3N2, and influenza B, only in 2009. A 95% confidence interval (CI) was estimated using simple exact binomial CIs. All analyses were performed using R 2·14·0 (R Foundation for Statistical Computing, Vienna, Austria).

## Results

### Influenza-like illness in the outpatient population

**Figure 2:**
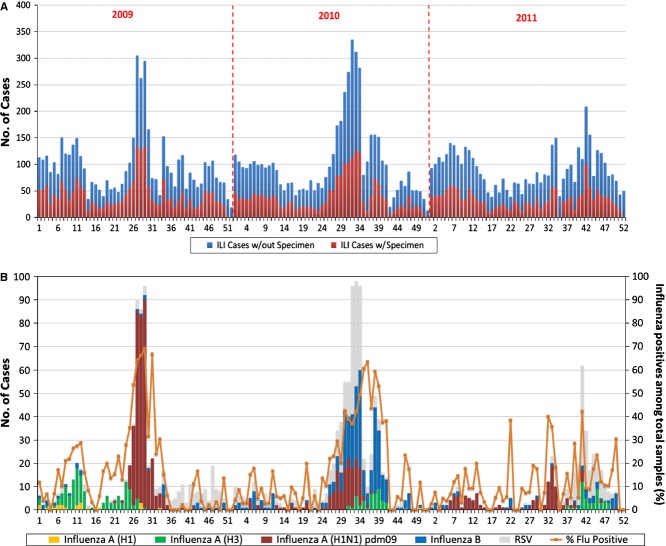
The number of patients with ILI whose specimens were obtained and the total number of patients with ILI in health centers by epidemiological week (A). The weekly number of positive cases for each targeted virus, with the percentage of positive influenza cases (B) in Baguio City from January 2009 to December 2011. ILI, influenza-like illness.

From January 2009 to December 2011, a total of 71 524 health center consultations were conducted and 13 002 (18·2%) ILI cases were identified. There were two peaks in ILI consultations, from January to March and from July to September of each year (Figure 2).

We obtained 5915 specimens from 13 002 ILI cases (45·5%). The mean virus detection rate for the 3-year period was 33%, while the influenza detection rate was 25%. The H1N1pdm09 positivity rate was 17% in 2009; however, this rate declined to 8% and 7% in 2010 and 2011, respectively. H3N2 and influenza B were continuously detected during the 3 years, with varying rates of positivity. The influenza B positivity rate was particularly high in 2010 (15·7%). Respiratory syncytial virus appeared to make a considerable contribution to the ILI cases: 7%, 15%, and 11% in 2009, 2010, and 2011, respectively (Table 1).

**Table 1:** The number of samples collected, the number of positive cases for each virus, and the viral positivity rates among influenza-like illness (ILI) cases by age group during 2009–2011

	ILI cases with specimens	Influenza A (H1N1)	Influenza A (H3N2)	Influenza A (H1N1) pdm09	Influenza B	Respiratory syncytial virus
2009						
Total	2156	22	136	359	33	157
<6 months	420	2 (9·1)	9 (6·6)	7 (1·9)	2 (6·1)	13 (8·3)
6–23 months	973	9 (40·9)	29 (21·3)	53 (14·8)	6 (18·2)	71 (45·2)
2–4 years	397	8 (36·4)	42 (30·9)	80 (22·3)	4 (12·1)	44 (28·0)
5–14 years	195	1 (4·5)	39 (28·7)	155 (43·2)	11 (33·3)	21 (13·4)
15–49 years	150	2 (9·1)	16 (11·8)	60 (16·7)	9 (27·3)	7 (4·5)
>50 years	21	0	1 (0·7)	4 (1·1)	1 (3·0)	1 (0·6)
2010						
Total	2022	1	48	152	317	304
<6 months	355	0	1 (2·1)	2 (1·3)	5 (1·6)	20 (6·6)
6–23 months	963	0	17 (35·4)	24 (15·8)	51 (16·1)	140 (46·1)
2–4 years	400	0	16 (33·3)	36 (23·7)	93 (29·3)	101 (33·2)
5–14 years	157	1 (100)	9 (18·8)	72 (47·4)	133 (42·0)	34 (11·2)
15–49 years	133	0	5 (10·4)	18 (11·8)	34 (10·7)	7 (2·3)
>50 years	14	0	0	0	1 (0·3)	2 (0·7)
2011						
Total	1737	0	47	120	47	188
<6 months	310	0	0	2 (1·7)	1 (2·0)	4 (2·1)
6–23 months	858	0	9 (19·1)	22 (18·3)	8 (16·3)	90 (47·9)
2–4 years	341	0	15 (31·9)	31 (25·8)	14 (28·6)	73 (38·8)
5–14 years	104	0	17 (36·2)	43 (35·8)	19 (38·8)	17 (9·0)
15–49 years	111	0	4 (8·5)	21 (17·5)	7 (14·3)	3 (1·6)
>50 years	13	0	2 (4·3)	1 (0·8)	0	1 (0·5)

The figure in parentheses is the proportion of positive cases in each age group.

Generally, the proportion of cases positive for each virus was higher among children aged <5 years throughout the 3-year period. There was also a certain proportion of positive cases, particularly H1N1pdm09- and influenza B-positive cases, among children aged 5–14 years. A low proportion of viral positivity was observed in ILI cases aged >50 years, but the number of tested samples was very small in this age group.

The incidence rates of targeted viruses among the ILI cases per 1000 individuals are shown in Table 2. Overall, we estimated 5·4 per 1000 (95% CI, 1·83–12·7) individuals as the mean incidence rate of influenza, including H1N1pdm09 influenza, among the outpatients. Among influenza viruses, H1N1pdm09 exhibited the highest incidence rate (3·50 per 1000) in 2009, which was almost equivalent in magnitude to the influenza B incidence rate in 2010 (3·31 per 1000). In 2011, the highest incidence rate (1·19 per 1000) was again estimated for H1N1pdm09, although this rate was much lower than that in 2009. Therefore, a different dominant virus/subtype was observed in each year. With regard to incidence rates by age group, higher rates were observed for all viruses except H1N1pdm09 among children aged 6–23 months, <6 months, or 2–4 years. Incidence rates among older age groups were significantly low, and those for influenza and RSV in cases aged >50 years were the lowest throughout the study period.

**Table 2:** Influenza incidence rates per 1000 individuals among outpatients by viruses/subtypes during 2009–2011

	Seasonal influenza	Influenza A (H1N1)*	Influenza A (H3N2)*	Influenza A (H1N1) pdm09	Influenza B*
2009					
Overall	1·86 (0·20–7·01)	0·21 (0–4·13)	1·33 (0·07–6·13)	3·50 (0·85–9·51)	0·32 (0–4·34)
<6 months	12·3 (6·44–21·4)	1·90 (0·21–7·06)	8·54 (3·81–16·5)	6·64 (2·59–13·9)	1·90 (0·21–7·06)
6–23 months	13·2 (7·05–22·4)	2·69 (0·49–8·30)	8·68 (3·90–16·7)	15·9 (9·05–25·8)	1·80 (0·18–6·90)
2–4 years	8·23 (3·60–16·1)	1·22 (0·05–5·95)	6·40 (2·44–13·6)	12·2 (6·34–21·2)	0·61 (0–4·88)
5–14 years	2·96 (0·60–8·71)	0·06 (0–3·81)	2·27 (0·33–7·64)	9·01 (4·12–17·1)	0·64 (0–4·93)
15–49 years	0·49 (0–4·66)	0·04 (0–3·76)	0·29 (0–4·28)	1·09 (0·04–5·73)	0·16 (0–4·02)
>50 years	0·29 (0–4·27)	0	0·14 (0–3·98)	0·57 (0–4·81)	0·14 (0–3·98)
2010					
Overall		0·01 (0–3·71)	0·50 (0–4·68)	1·59 (0·13–6·56)	3·31 (0·76–9·23)
<6 months		0	1·24 (0·06–5·99)	2·49 (0·41–7·99)	6·22 (2·34–13·4)
6–23 months		0	5·66 (2·0–12·6)	7·99 (2·45–15·8)	17·0 (9·89–27·2)
2–4 years		0·06 (0–3·69)	2·51 (0·42–8·02)	5·64 (1·99–12·6)	14·6 (8·08–24·2)
5–14 years		0	0·53 (0–4·73)	4·23 (1·21–10·6)	7·82 (3·34–15·5)
15–49 years		0	0·10 (0–3·90)	0·37 (0–3·69)	0·70 (0–5·03)
>50 years		0	0	0	0·17 (0–4·05)
2011					
Overall		0	0·47 (0–4·61)	1·19 (0·05–5·90)	0·49 (0–4·65)
<6 months		0	0	2·11 (0·28–7·39)	1·05 (0·03–5·66)
6–23 months		0	2·68 (0·49–8·28)	6·55 (2·54–13·8)	2·38 (0·37–7·82)
2–4 years		0	2·32 (0·35–7·73)	4·80 (1·51–11·4)	2·17 (0·3–7·49)
5–14 years		0	1·0 (0·03–5·58)	2·54 (0·43–8·06)	1·12 (0·04–5·78)
15–49 years		0	0·08 (0–3·86)	0·43 (0–4·55)	0·14 (0–3·99)
>50 years		0	0·47 (0–4·61)	0·15 (0–4·01)	0

The figures in parentheses are 95% confidence intervals.

* The incidence rate of seasonal influenza included cases positive for influenza A(H1), A(H3), and B.

### Severe acute respiratory infection in the inpatient population

**Figure 3:**
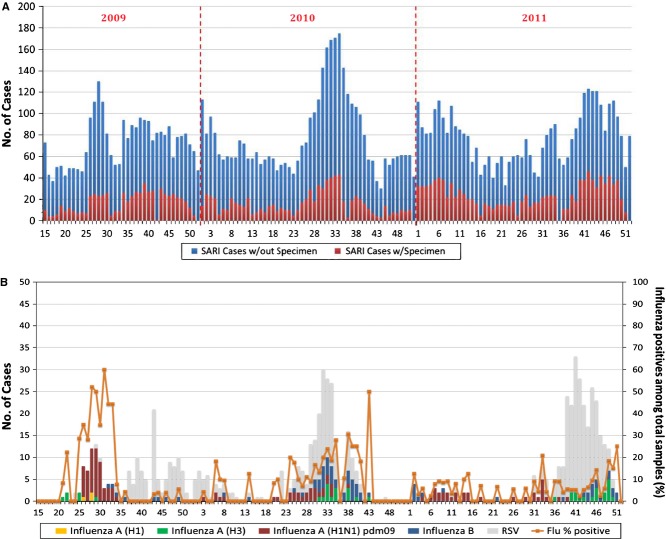
The number of patients with sARI whose specimens were obtained and the total number of patients with sARI in hospitals by morbidity week (A). The weekly number of positive cases for each targeted virus, with the percentage of positive influenza cases (B) in Baguio City from April 2009 to December 2011. sARI, severe acute respiratory infection.

From April 2009 to December 2011, 10 726 (12·8%) of 83 667 cases admitted to five hospitals were diagnosed with sARI on admission. Totally, 2656 specimens (24·8%) were obtained. Severe acute respiratory infection appeared to be a common cause of hospital confinement during the 3-year period, with a prominent peak from July to September (Figure 3). The highest proportion (50·8%) of children admitted for sARI belonged to the <5-year age group.

The overall and influenza virus detection rates during the period were 29% and 9%, respectively. Respiratory syncytial virus was more common than influenza virus among the sARI cases, particularly among children aged <2 years, throughout the study period (Table 3). Different types of influenza viruses were detected in the 3 years, but its frequencies fluctuated with virus types and study years. There was only one positive case of seasonal H1N1 in 2009.

**Table 3:** The number of samples collected, the number of positive cases for each virus, and the viral positivity rates among severe acute respiratory infection (sARI) cases by age group during 2009–2011

	sARI cases with specimens	Influenza A (H1N1)	Influenza A (H3N2)	Influenza A (H1N1) pdm09	Influenza B	Respiratory syncytial virus
2009						
Total	626	1	8	53	8	105
<6 months	84	0	0	4 (7·5)	1 (12·5)	31 (29·5)
6–23 months	222	1 (100)	3 (37·5)	9 (17·0)	4 (50·0)	53 (50·5)
2–4 years	73	0	1 (12·5)	7 (13·2)	1 (12·5)	12 (11·4)
5–14 years	70	0	1 (12·5)	17 (32·1)	1 (12·5)	4 (3·8)
15–49 years	84	0	2 (25·0)	10 (18·9)	1 (12·5)	3 (2·9)
>50 years	93	0	1 (12·5)	6 (11·3)	0	2 (1·9)
2010						
Total	812	0	15	27	41	188
<6 months	154	0	1 (6·7)	3 (11·1)	3 (7·3)	59 (31·4)
6–23 months	258	0	2 (13·3)	5 (18·5)	8 (19·5)	89 (47·3)
2–4 years	117	0	2 (13·3)	3 (11·1)	8 (19·5)	23 (12·2)
5–14 years	78	0	1 (6·7)	4 (14·8)	16 (39·0)	7 (3·7)
15–49 years	102	0	8 (53·3)	7 (25·9)	5 (12·2)	5 (2·7)
>50 years	103	0	1 (6·7)	5 (18·5)	1 (2·4)	5 (2·7)
2011						
Total	1218	0	19	31	23	241
<6 months	186	0	2 (10·5)	2 (6·5)	1 (4·3)	72 (29·9)
6–23 months	410	0	7 (36·8)	11 (35·5)	5 (21·7)	113 (46·9)
2–4 years	215	0	3 (15·8)	3 (9·7)	5 (21·7)	36 (14·9)
5–14 years	118	0	3 (15·8)	2 (6·5)	3 (13·0)	7 (2·9)
15–49 years	110	0	2 (10·5)	7 (22·6)	3 (13·0)	4 (1·7)
>50 years	179	0	2 (10·5)	6 (19·4)	6 (26·1)	9 (3·7)

The figure in parentheses is the proportion of positive cases in each age group.

Twenty-six deaths were recorded among sARI cases during the 3-year period. Fourteen were males, 11 were aged ≥60 years, and seven were aged <5 years. All mortalities except one were clinically diagnosed with pneumonia, but only three were positive for viruses in all victims; one male aged 40 years was positive for H1N1pdm09, and two infants were positive for RSV.

Overall, 1·0 per 1000 individuals (95% CI, 0·03–5·57) was estimated as the mean incidence rate of sARI associated with influenza, including H1N1pdm09 (Table 4). The highest sARI incidence rates were observed in association with H1N1pdm09 in 2009, followed by influenza B in 2010 and H1N1pdm09 again in 2011. The influenza A and B incidence rate was highest among children aged 6–23 months in all years except 2009 and 2010, when the H1N1pdm09 influenza incidence rate was the highest. H1N1pdm09 influenza posed a certain burden across all age groups in 2009, but the incidence rate significantly decreased in the following years among all age groups except the above 50-year age group, in which almost all incidence rates remained constant throughout. The influenza B incidence rate was 0·91 per 1000 individuals aged >50 years in 2011, whereas none of the outpatients were reported to belong to this age group through ILI surveillance (Table 2).

**Table 4:** Influenza incidence rates per 1000 individuals among severe acute respiratory infection (sARI) cases by viruses/subtypes during 2009–2011

	Seasonal influenza	Influenza A (H1N1)*	Influenza A (H3N2)*	Influenza A (H1N1) pdm09	Influenza B*
2009					
Overall	0·30 (0–4·29)	0·02 (0–3·73)	0·14 (0–3·98)	0·93 (0·02–5·45)	0·14 (0–3·98)
<6 months	1·40 (0·09–6·25)	0	0	4·33 (1·26–10·7)	1·08 (0·03–5·71)
6–23 months	3·68 (0·93–9·78)	0·35 (0–4·39)	1·05 (0·03–5·66)	3·15 (0·68–8·99)	1·40 (0·09–6·25)
2–4 years	0·69 (0–5·02)	0	0·29 (0–4·28)	2·04 (0·25–7·28)	0·29 (0–4·28)
5–14 years	0·20 (0–4·09)	0	0·08 (0–3·86)	1·37 (0·08–6·20)	0·08 (0–3·86)
15–49 years	0·09 (0–3·88)	0	0·05 (0–3·80)	0·26 (0–4·21)	0·03 (0–3·74)
Over 50 years	0·23 (0–4·15)	0	0·20 (0–4·09)	1·19 (0·02–5·45)	0
2010					
Overall		0	0·29 (0–4·27)	0·52 (0·00–4·70)	0·79 (0·01–5·19)
<6 months		0	0·81 (0·01–5·23)	2·42 (0·39–7·88)	2·42 (0·39–7·88)
6–23 months		0	0·81 (0·01–5·23)	2·02 (0·25–7·25)	3·22 (0·72–9·10)
2–4 years		0	0·58 (0–4·81)	0·86 (0·01–5·33)	2·30 (0·34–7·70)
5–14 years		0	0·12 (0–3·93)	0·46 (0–4·60)	1·85 (0·20–6·99)
15–49 years		0	0·23 (0–4·15)	0·20 (0–4·09)	0·14 (0–3·98)
Over 50 years		0	0·27 (0–4·24)	1·35 (0·08–6·17)	0·27 (0–4·24)
2011					
Overall		0	0·25 (0–4·19)	0·40 (0·00–4·49)	0·30 (0–4·29)
<6 months		0	1·35 (0·08–6·16)	1·35 (0·08–6·16)	0·67 (0–4·99)
6–23 months		0	1·83 (0·19–6·96)	2·88 (0·57–8·59)	1·31 (0·07–6·10)
2–4 years		0	0·45 (0–4·59)	0·45 (0–4·59)	0·76 (0·01–5·14)
5–14 years		0	0·18 (0–4·06)	0·12 (0–3·94)	0·18 (0–4·06)
15–49 years		0	0·04 (0–3·77)	0·14 (0–3·98)	0·06 (0–3·81)
Over 50 years		0	0·30 (0–4·30)	0·91 (0·02–5·41)	0·91 (0–4·29)

The figures in parentheses are 95% confidence intervals.

* Seasonal influenza incidence rates included positive cases of influenza A (H1), A (H3), and B.

## Discussion

During the entire study period, the overall influenza-positive rate among medically attended ILI cases was 25%; this is similar to findings of studies in neighboring countries.^11^–^13^ Among the hospitalized cases, the positivity rate for influenza was 9%, and 49% of these cases were attributed to H1N1pdm09. These positivity rates are also similar to those found in other tropical countries such as Kenya,^14^ Bangladesh,^15^ and Thailand.^16^ Several factors affect virus positivity rate, such as demographic characteristics and health-seeking behavior of the study population, differences in sample storage and testing methods, and duration between onset and consultation. Despite these varying factors, our study demonstrated influenza positivity rates that were equivalent to those estimated in other countries, indicating that there was a certain influenza burden in both outpatient and inpatients.

Besides year-round influenza activities, there appear to be two peaks in each year, generally from February to March and June to September–December. We found that the latter peaks in each year were more distinct, and those periods were usually recognized as a rainy season in Baguio city. The peaks of influenza during rainy season were documented in other tropical and subtropical countries;^17^–^20^ however, our study is unique in observing above tendency in the cooler temperature. As a pooled study demonstrated,^21^ precipitation can play an important role in epidemics of influenza especially in tropics partly because people change their behaviors during rainy seasons.^22^ All peaks except that due to H1N1pdm09 observed in 2009 were caused by multiple viruses. The peak due to H1N1pdm09 in 2009 was observed as similar timing in Thailand^17^ and Lao PDR,^12^ and earlier than that in Vietnam^23^ and Taiwan.^24^ Peaks in Lao PDR and Taiwan consisted of H1N1pdm09 and influenza A(H3N2). Another peak observed in 2010 consisted of influenza B, H1N1pdm09, and influenza A(H3N2). The combination of these viruses were also reported in those countries in August of 2010, but dominant virus in peak was different influenza B in Lao PDR, H1N1pdm09 in Taiwan and Thailand, influenza A(H3N2) in Vietnam. A pooled study in Western Pacific region illustrated a similar temporal distribution of influenza except the absence of influenza A(H3N2) and H1N1pdm09 in 2010.^25^

In other countries, the reported incidences of H1N1pdm09 influenza are significantly higher than those of seasonal influenza.^25^,^26^ In our study, however, the H1N1pdm09 influenza incidence rate in 2009 was comparable with the influenza B incidence rate in 2010, although baseline seasonal influenza rates were unknown because the present study did not cover years prior to 2009. The peak due to H1N1pdm09 was about twofold higher than that due to influenza B in 2010, while the number of weeks when those viruses were detected was twofold higher in influenza B compared with H1N1pdm09. Thus, H1N1pdm09 posed larger peak within a short interval compared with influenza B. Moreover, the impact of H1N1pdm on hospitalization was larger than that of seasonal influenza during the 2009 outbreak reported from other countries.^27^–^29^ However, the present study did not also show a significant impact in 2009. The reason for the low number of hospitalizations attributed to H1N1pdm09 in 2009 remains unclear.

With regard to influenza incidence rates across age groups, both outpatient and inpatient children aged <5 years exhibited the highest incidence rates of 22·6 per 1000 (95% CI, 14·3–34·0) and 4·65 per 1000 individuals (95% CI, 1·43–11·2), respectively. These estimates are compatible with the estimated incidence rates among outpatients in similar studies^15^,^30^ and also with global estimated sARI incidence rates.^31^ While the outpatients' incidence rates were compatible between patients aged <2 and 2–4 years, those among inpatients were two- to threefold higher in the <2-year age group. The difference in hospitalization rates between these age groups has been documented elsewhere.^2^,^31^–^33^ Our data added the finding that influenza could cause a significant hospitalization among children aged <2 years. The incidence rate was low among adult outpatients of all age groups, whereas that was higher for adult inpatients aged >50 years than for adult inpatients aged 20–49 years. This could be partly explained by the fact that city health centers are the first level of medical facility and provide primary care mainly for children. In addition, sick adults and elderly individuals may possibly behave differently; although self-medication and rest may be the primary treatment for most ILI episodes, any aggravation leads them either to specialized outpatient department of hospitals or private clinics, particularly with the presence of other complications. Therefore, we need to carefully consider the incidence of outpatients and inpatients among adults while discussing the entire influenza disease burden in the country.

In developed countries, significant effects of mortality from seasonal influenza among the elderly have been well documented.^34^,^35^ However, the mortality impact of influenza in developing countries is not fully elucidated. We identified only one case of mortality in an adult with H1N1pdm09 influenza. This low mortality rate can be explained by several reasons. First, there is a possibility that many sARI cases in the study population may not have been hospitalized. In a study from Bangladesh, only 11% patients who died within 2 weeks of developing ILI died in hospitals.^36^ Second, mortality rates also depend on patient age. In this study, 64% sARI patients whose samples were obtained were aged <5 years, while only 3·8% were aged ≥65 years. Third, the limited degree of influenza testing could have led to the underestimation of influenza-associated deaths. It is also possible that the mortality impact of influenza is actually low. The proportion of the elderly, especially those with severe chronic medical conditions, is relatively low in developing countries. This demographic characteristic may affect the overall mortality impact of influenza. Data on mortality impact are particularly important in developing any national control strategy for influenza, including a vaccination policy. Further studies are necessary to define the mortality impact of influenza in the country.

This study had some limitations. This study measured rates of medically attended influenza, which represented a subset of the overall community burden as not all people with influenza consulted for their illnesses. Additionally, rates of medically attended influenza could be underestimated for the following reasons; not all people with medically attended influenza met the ILI case definition, and these cases would be missed by the surveillance. Second, the surveillance did not cover private clinics, and thus, it was a chance to miss the ILI cases. Third, because actual surveillance days at the health facilities are fixed weekly day, viral and influenza detection rates were extrapolated to the remaining non-surveillance days of the week. Fourth, in sARI surveillance, refusal rates were progressively increasing, which may affect the estimation of sARI incidence in the study. However, the trends of influenza associated with both ILI and sARI were similar, and both positivity and incidence rates were compatible with those in other studies; therefore, we believe that our findings can define the influenza burden among medically attended cases.

In conclusion, we observed a year-round influenza activity during 2009–2011 in Baguio city. Higher influenza incidence rates were observed particularly among those aged <2 years; therefore, these should be the focus of public health action. Influenza incidence and hospitalization rates are good indicators for estimating disease burden in countries where influenza-associated mortality appears to be low. There is a need to establish a study that covers the general population in the community to better understand influenza disease burden, which is a key for developing further influenza control policies such as vaccine administration.

## Addendum

Conceived, designed, and supervised surveillance: VLT, TK, RRP, HO, and RMO. Performed PCR testing: ESM; developed the database: PPA, AGT, JBJ; analyzed the data: VLT, TK, AGT; wrote the paper: VLT, TK; revised and approved final version of the manuscript: VLT, TK, AGT, RRP, PPA, ESM, JBJ, HO, and RMO.

## Financial support

This work was supported by the United States Centers for Disease Control and Prevention (US-CDC) under US-CDC Cooperative Agreement [U50/CCU2444, 5U5IIP000335] and by a grant-in-aid from the Japan Initiative for Global Research Network on Infectious Diseases, Ministry of Education, Culture, Sports, Science and Technology, Japan.

## References

[1] Thompson WW, Shay DK, Weintraub E (2003). Mortality associated with influenza and respiratory syncytial virus in the United States. JAMA.

[2] Chiu SS, Lau YL, Chan KH, Wong WH, Peiris JS (2002). Influenza-related hospitalizations among children in Hong Kong. N Engl J Med.

[3] Zucs P, Buchholz U, Haas W, Uphoff H (2005). Influenza associated excess mortality in Germany, 1985–2001. Emerg Themes Epidemiol.

[4] Simmerman JM, Uyeki TM (2008). The burden of influenza in East and South-East Asia: a review of the English language literature. Influenza Other Respir Viruses.

[5] The World Bank (2011). World Development Indicators 2011.

[6] WHO Regional Office for the Western Pacific (2011). Western Pacific Country Health information Profiles: 2010 Revision.

[7] WHO (2012). Recommendations for management of common childhood conditions, Evidence for technical update of pocket book recommendations.

[8] (2009).

[10] Bellau-Pujol S, Vabret A, Legrand L (2005). Development of three multiplex RT-PCR assays for the detection of 12 respiratory RNA viruses. J Virol Methods.

[11] Nguyen HT, Dharan NJ, Le MT (2009). National influenza surveillance in Vietnam, 2006–2007. Vaccine.

[12] Khamphaphongphane B, Ketmayoon P, Lewis HC (2012). Epidemiological and virological characteristics of seasonal and pandemic influenza in Lao PDR, 2008–2010. Influenza Other Respir Viruses.

[13] Blair PJ, Wierzba TF, Touch S (2010). Influenza epidemiology and characterization of influenza viruses in patients seeking treatment for acute fever in Cambodia. Epidemiol Infect.

[14] Feikin DR, Ope MO, Aura B (2012). The population-based burden of influenza-associated hospitalization in rural western Kenya, 2007–2009. Bull World Health Organ.

[15] Azziz-Baumgartner E, Alamgir AS, Rahman M (2012). Incidence of influenza-like illness and severe acute respiratory infection during three influenza seasons in Bangladesh, 2008–2010. Bull World Health Organ.

[16] Simmerman JM, Chittaganpitch M, Levy J (2009). Incidence, seasonality and mortality associated with influenza pneumonia in Thailand: 2005–2008. PLoS ONE.

[17] Chittaganpitch M, Supawat K, Olsen SJ (2012). Influenza viruses in Thailand: 7 years of sentinel surveillance data, 2004–2010. Influenza Other Respir Viruses.

[18] Moura FE, Perdigao AC, Siqueira MM (2009). Seasonality of influenza in the tropics: a distinct pattern in northeastern Brazil. Am J Trop Med Hyg.

[19] Dapat C, Saito R, Kyaw Y (2009). Epidemiology of human influenza A and B viruses in Myanmar from 2005 to 2007. Intervirology.

[20] Zaman RU, Alamgir AS, Rahman M (2009). Influenza in outpatient ILI case-patients in national hospital-based surveillance, Bangladesh, 2007–2008. PLoS ONE.

[21] Tamerius JD, Shaman J, Alonso WJ (2013). Environmental predictors of seasonal influenza epidemics across temperate and tropical climates. PLoS Pathog.

[22] Murray EL, Klein M, Brondi L (2012). Rainfall, household crowding, and acute respiratory infections in the tropics. Epidemiol Infect.

[23] Nguyen YT, Graitcer SB, Nguyen TH (2013). National surveillance for influenza and influenza-like illness in Vietnam, 2006–2010. Vaccine.

[24] Chuang JH, Huang AS, Huang WT (2012). Nationwide surveillance of influenza during the pandemic (2009–10) and post-pandemic (2010–11) periods in Taiwan. PLoS ONE.

[25] Members of the Western Pacific Region Global Influenza Surveillance and Response System (2012). Epidemiological and virological characteristics of influenza in the Western Pacific Region of the World Health Organization, 2006–2010. PLoS ONE.

[26] Van Kerkhove MD, Mounts AW, Mall S (2011). Epidemiologic and virologic assessment of the 2009 influenza A (H1N1) pandemic on selected temperate countries in the Southern Hemisphere: Argentina, Australia, Chile, New Zealand and South Africa. Influenza Other Respir Viruses.

[27] Libster R, Bugna J, Coviello S (2010). Pediatric hospitalizations associated with 2009 pandemic influenza A (H1N1) in Argentina. N Engl J Med.

[28] Cox CM, D'Mello T, Perez A (2012). Increase in rates of hospitalization due to laboratory-confirmed influenza among children and adults during the 2009–10 influenza pandemic. J Infect Dis.

[29] Bishop JF, Murnane MP, Owen R (2009). Australia's winter with the 2009 pandemic influenza A (H1N1) virus. N Engl J Med.

[30] Poehling KA, Edwards KM, Weinberg GA (2006). The underrecognized burden of influenza in young children. N Engl J Med.

[31] Nair H, Brooks WA, Katz M (2011). Global burden of respiratory infections due to seasonal influenza in young children: a systematic review and meta-analysis. Lancet.

[32] Grijalva CG, Craig AS, Dupont WD (2006). Estimating influenza hospitalizations among children. Emerg Infect Dis.

[33] Chiu SS, Chan KH, Chen H (2009). Virologically confirmed population-based burden of hospitalization caused by influenza A and B among children in Hong Kong. Clin Infect Dis.

[34] Lee VJ, Yap J, Ong JB (2009). Influenza excess mortality from 1950–2000 in tropical Singapore. PLoS ONE.

[35] Yang L, Ma S, Chen PY (2011). Influenza associated mortality in the subtropics and tropics: results from three Asian cities. Vaccine.

[36] Homaira N, Luby SP, Alamgir AS (2012). Influenza-associated mortality in 2009 in four sentinel sites in Bangladesh. Bull World Health Organ.

